# Supplementary material for: Anterior cingulate cross-hemispheric inhibition via the claustrum resolves painful sensory conflict
Source: Commun Biol. 2024 Mar 15;7:330. doi: 10.1038/s42003-024-06008-9 (PMC10943010; doi:10.1038/s42003-024-06008-9)
Supplement: Supplementary file 2 — Supplementary information [file 42003_2024_6008_MOESM2_ESM.pdf]

Supplementary Information

Anterior cingulate cross-hemispheric inhibition via the claustrum resolves painful sensory conflict.

Keisuke Koga, Kenta Kobayashi, Makoto Tsuda, Anthony E Pickering, Hidemasa Furue

This PDF file includes

Supplementary Figure 1 to 10

Legends for Supplementary Figures

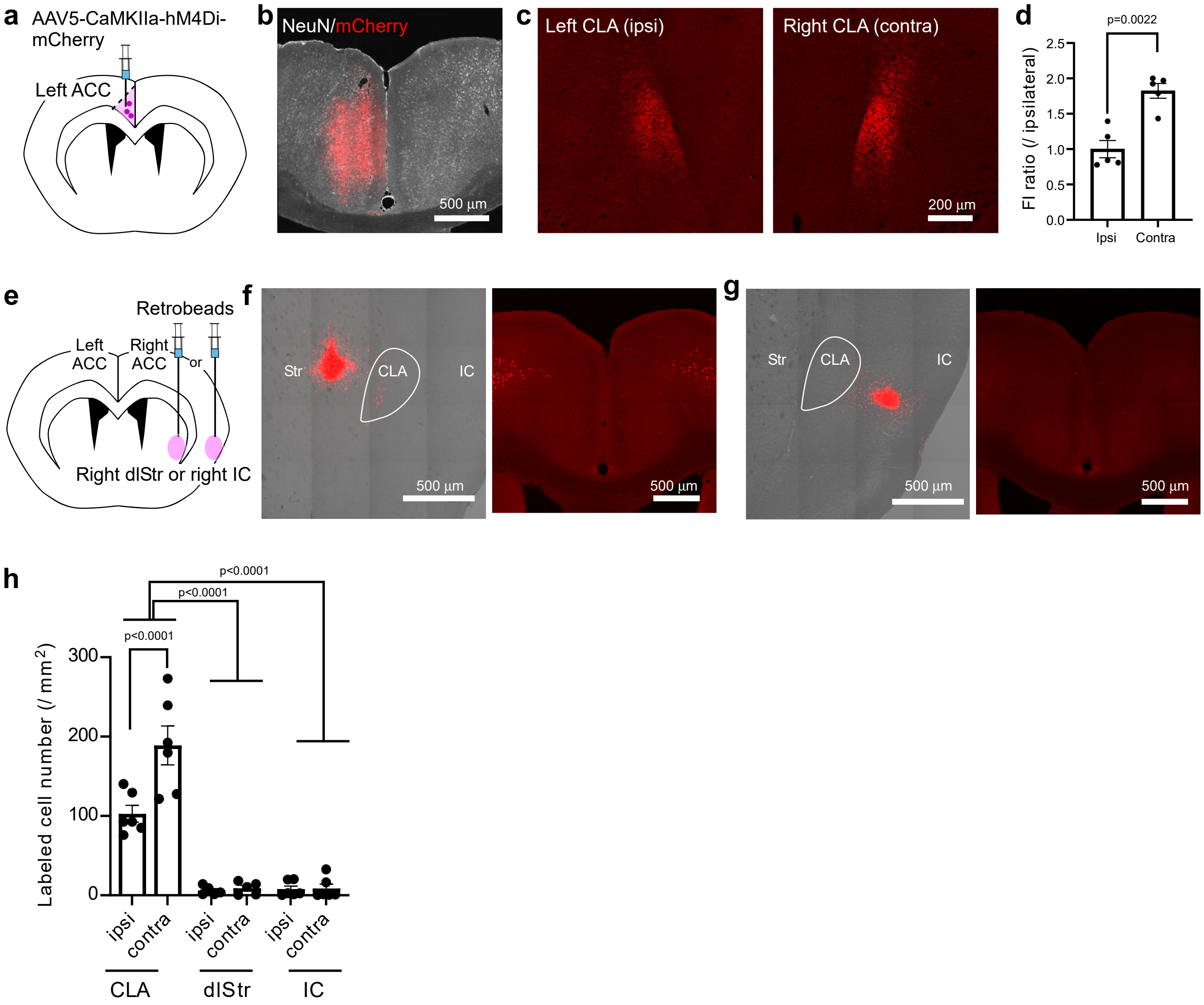

a
AAV5-CaMKIIa-hM4Di-mCherry
Left ACC
b
NeuN/mCherry
500 µm
c
Left CLA (ipsi)
Right CLA (contra)
200 µm
d
p=0.0022
FI ratio (/ ipsilateral)
Ipsi
Contra
e
Retrobeads
Left ACC
Right ACC
or
Right dlStr or right IC
f
Str
CLA
IC
500 µm
g
Str
CLA
IC
500 µm
h
p<0.0001
p<0.0001
p<0.0001
Labeled cell number (/ mm²)
ipsi
contra
CLA
dlStr
IC

Supplementary Figure 1. ACC projections to the adjacent insular cortex (IC) or dorsolateral striatum (dlStr) were much fewer than the projections to the claustrum.

a Schematic of AAV (AAV5-CaMKIIα-hM4Di-mCherry) injection into the left ACC. **b** Representative coronal brain sections showing mCherry expression (red) of the pyramidal neurons in the ACC. **c** Representative images of terminals of the ACC pyramidal neurons (enhanced by a RFP antibody) in the left (ipsilateral to the AAV injection side) and the right (contralateral) CLA. **d** Quantification of the fluorescent intensity (FI) of the contralateral mCherry-expressing terminals and that of the ipsilateral terminals (n = 5, two-tailed paired t-test). **e–g** Retrograde tracing from the IC or dlStr. Schematic of the retrograde tracer injections (**e**). Representative coronal sections showing Retrobeads injection into the right dlStr (*left*) and its Retrobead-labeled (bead^{+}) cells in both side of the ACC (*right*) (**f**). Those of Retrobeads injection into the right IC (**g**). **h** Quantification of the number of bead^{+} ACC neurons in the right (ipsilateral to the Retrobeads injection side) and left (contralateral) ACC (CLA, n = 6 mice; dlStr n = 5 mice; IC n = 6 mice, two-way repeated measures ANOVA with Bonferroni's multiple comparisons test). Error bars show the SEM.

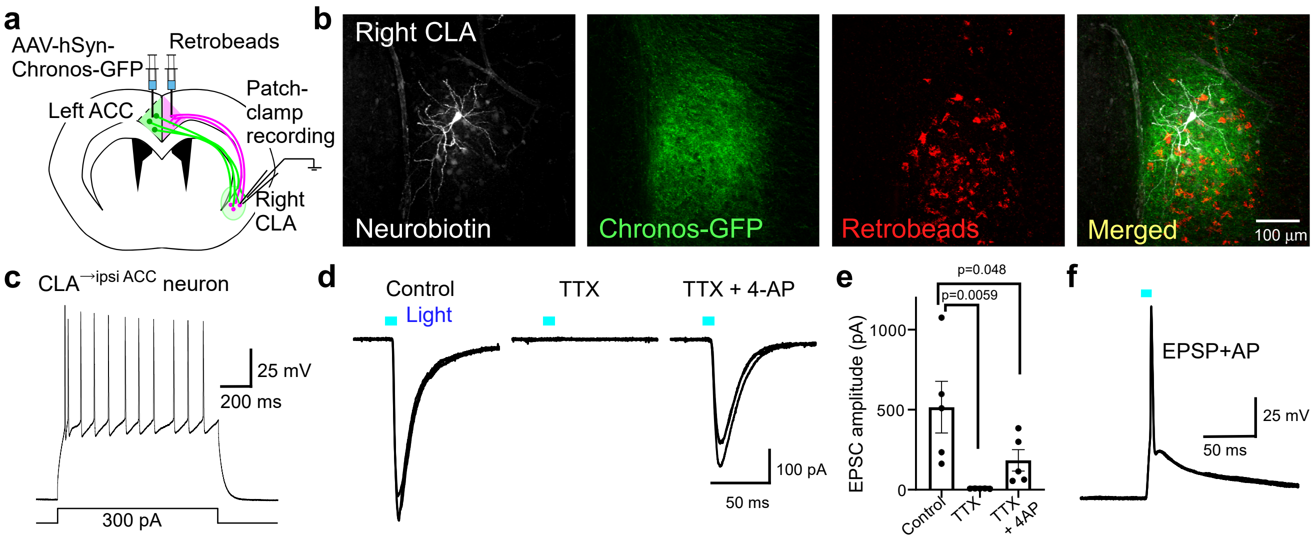

a
AAV-hSyn-Chronos-GFP
Retrobeads
Left ACC
Patch-clamp recording
Right CLA
b
Right CLA
Neurobiotin
Chronos-GFP
Retrobeads
Merged
100 μm
c
CLA→ipsi ACC neuron
25 mV
200 ms
300 pA
d
Control
Light
TTX
TTX + 4-AP
100 pA
50 ms
e
p=0.048
p=0.0059
EPSC amplitude (pA)
1000
500
0
Control
TTX
TTX + 4AP
f
EPSP+AP
25 mV
50 ms

Supplementary Figure 2. ACC neurons monosynaptically contact with contralateral CLA$^{\rightarrow\mathrm{ipsiACC}}$ neurons.

a Schematic of injections of AAV (Chronos-GFP) vector into the left ACC and Retrobeads into the right ACC, and slice patch-clamp recording from retrogradely labelled bead^{+} CLA$^{\rightarrow\mathrm{ipsiACC}}$ neurons. **b** Representative images of the right CLA showing a recorded bead^{+} CLA$^{\rightarrow\mathrm{ipsiACC}}$ neuron stained with neurobiotin in the recording pipette (neurobiotin, gray; Chronos-GFP, green; Retrobeads, red). **c**–**f** Firing pattern and synaptic responses in bead^{+} CLA$^{\rightarrow\mathrm{ipsiACC}}$ neurons. Representative firing pattern in response to depolarizing current (**c**), and EPSCs evoked by light stimulation of the Chronos-GFP expressing terminals of the left ACC neurons at a holding potential of -70 mV in control, in the presence of TTX (1 μM), and TTX + 4-AP (200 μM). We showed 3 traces for each condition. (**d**). Summary showing the EPSC amplitudes (**e**: n = 5, one-way repeated mesures ANOVA with Tukey's multiple comparisons test). An example of the light-evoked EPSP with an AP (**f**). Error bars show the SEM.

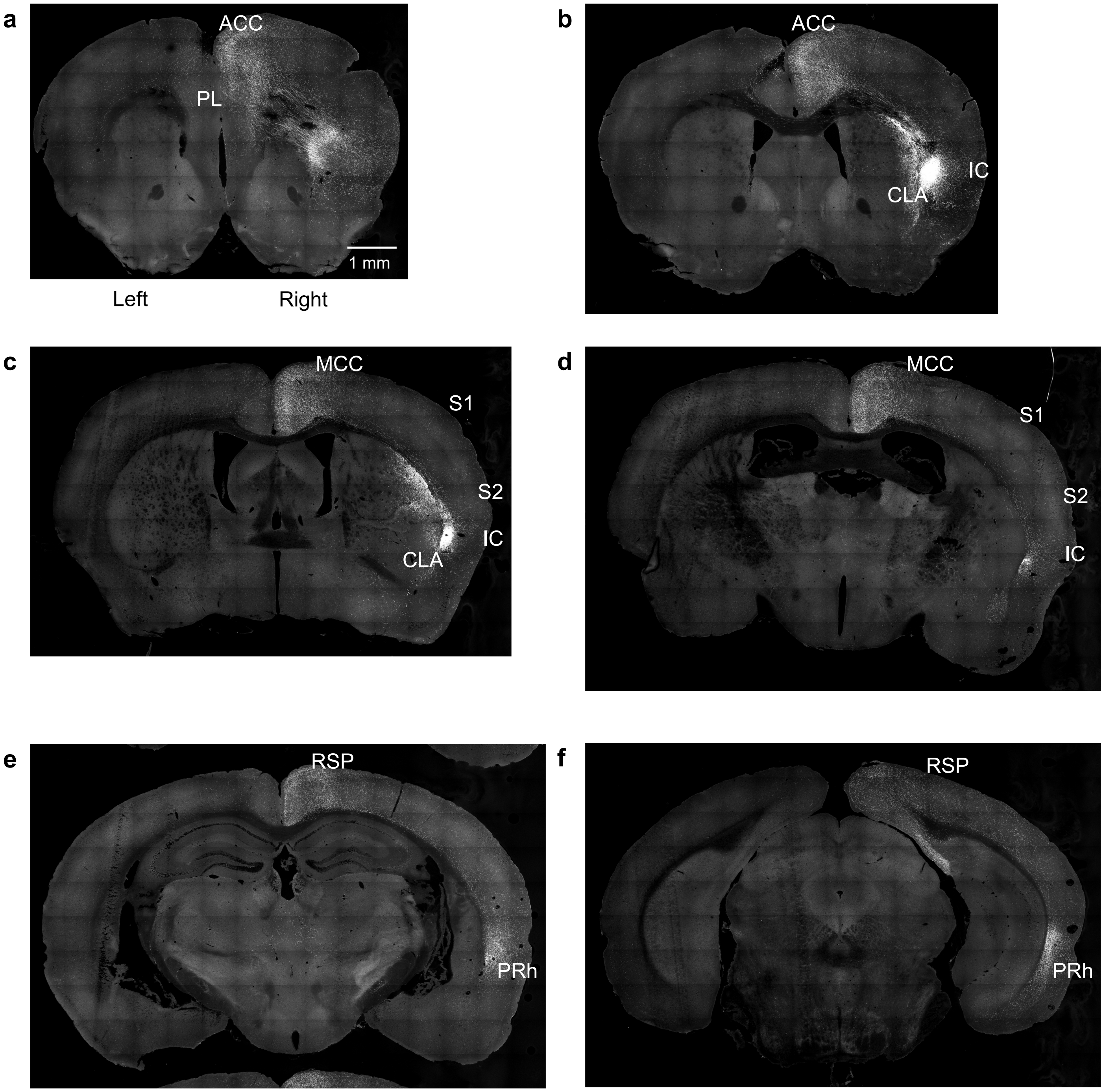
a
ACC
PL
1 mm
Left
Right
b
ACC
IC
CLA
c
MCC
S1
S2
IC
CLA
d
MCC
S1
S2
IC
e
RSP
PRh
f
RSP
PRh

Supplementary Figure 3. $CLA_{\leftarrow contraACC}$ neurons selectively project to the cingulate cortex. **a-f** Coronal brain sections showing anterograde tracing of $CLA_{\leftarrow contraACC}$ neurons. AAV1-EF1α-Flpo was injected into the left ACC followed by injection of a Flp-dependent Chronos-GFP expression virus, AAV_{DJ}-fDIO-Chronos-GFP, into the right CLA. Anterior cingulate cortex (ACC), prelimbic cortex (PL), insular cortex (IC), midcingulate cortex (MCC), primary somatosensory cortex (S1), secondary somatosensory cortex (S2), retrosplenial cortex (RSP) and perirhinal cortex (PRh) .

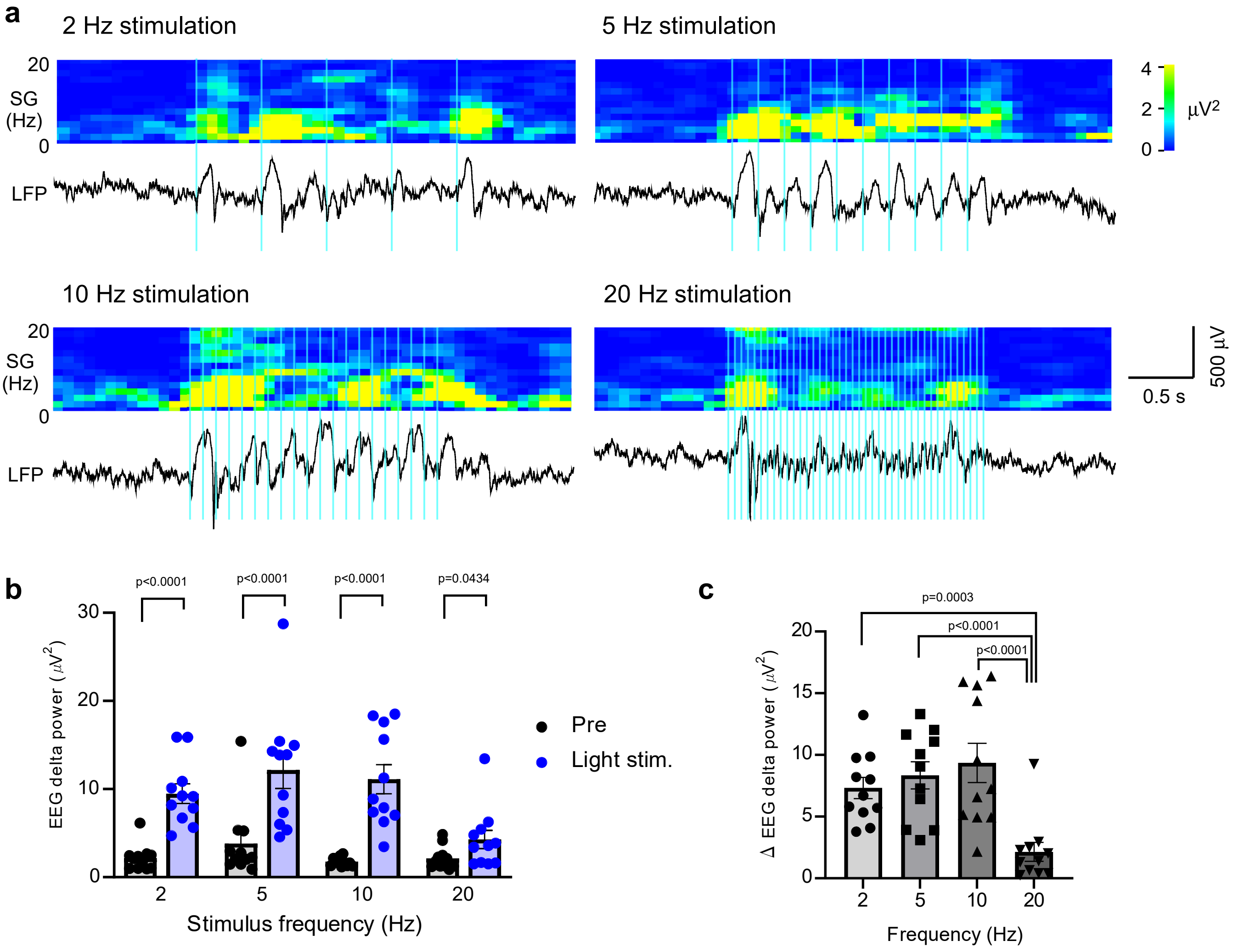

a
2 Hz stimulation
5 Hz stimulation
10 Hz stimulation
20 Hz stimulation
SG (Hz)
LFP
μV2
500 μV
0.5 s
b
p<0.0001
p<0.0001
p<0.0001
p=0.0434
EEG delta power (μV2)
Stimulus frequency (Hz)
Pre
Light stim.
c
p=0.0003
p<0.0001
p<0.0001
Δ EEG delta power (μV2)
Frequency (Hz)

Supplementary Figure 4. Optical activation of ACC neuronal terminals in the contralateral CLA facilitates the EEG delta power in the contralateral ACC in a frequency-dependent manner.

a Representative Spectrograms (SGs, top) and traces of the LFP signals (below). The timing of light-stimulation was indicated as blue dashed lines in each trace (2 Hz, 4 times; 5 Hz, 10 times; 10 Hz, 20 times; 20 Hz, 40 times). **b** Summary of EEG delta power in the right ACC before (control) and during each light stimulation (n = 11, for 2 s each, two-way repeated measures ANOVA with Bonferroni's multiple comparisons test). **c** Summary of EEG delta power differences between before (pre) and during light stimulation in right ACC (n = 11, for 2 s each, one-way repeated measures repeated measures ANOVA with Tukey's multiple comparisons test). Error bars show the SEM.

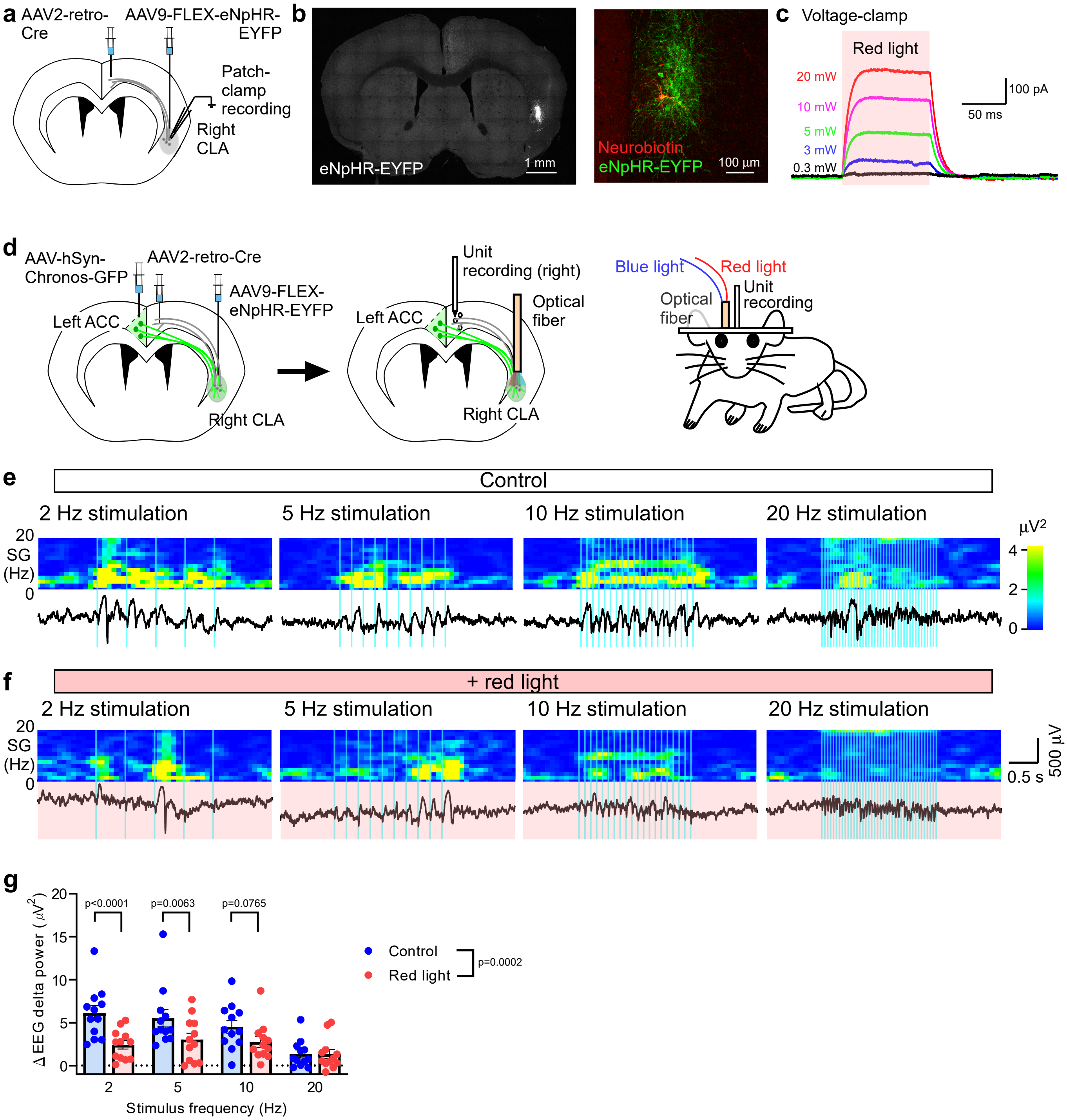

a
AAV2-retro-Cre
AAV9-FLEX-eNpHR-EYFP
Patch-clamp recording
Right CLA
b
eNpHR-EYFP
1 mm
Neurobiotin
eNpHR-EYFP
100 µm
c
Voltage-clamp
Red light
20 mW
10 mW
5 mW
3 mW
0.3 mW
100 pA
50 ms
d
AAV-hSyn-Chronos-GFP
AAV2-retro-Cre
AAV9-FLEX-eNpHR-EYFP
Left ACC
Right CLA
Unit recording (right)
Optical fiber
Left ACC
Right CLA
Blue light
Red light
Optical fiber
Unit recording
e
Control
2 Hz stimulation
5 Hz stimulation
10 Hz stimulation
20 Hz stimulation
20
SG (Hz)
0
µV2
4
2
0
f
+ red light
2 Hz stimulation
5 Hz stimulation
10 Hz stimulation
20 Hz stimulation
20
SG (Hz)
0
500 µV
0.5 s
g
Δ EEG delta power (µV2)
20
15
10
5
0
p<0.0001
p=0.0063
p=0.0765
2
5
10
20
Stimulus frequency (Hz)
Control
Red light
p=0.0002

Supplementary Figure 5. Optical inhibition of the contralateral CLA$^{\rightarrow \mathrm{ipsiACC}}$ neurons suppressed the EEG delta power increase in the contralateral ACC by the activation of ACC$^{\rightarrow \mathrm{contraCLA}}$ neurons.

a–c Schematic of a retrograde transduction strategy for eNpHR-YFP expression in the right CLA$^{\rightarrow \mathrm{ipsiACC}}$ neurons (**a**) and a representative image showing the eNpHR-YFP-expressing neurons (gray) (**b**, *left*). A recorded CLA$^{\rightarrow \mathrm{ipsiACC}}$ neuron stained with neurobiotin (**b**, *right image*; neurobiotin, red; eNpHR-YFP, green), and traces of outward currents recorded in voltage-clamp mode from the CLA$^{\rightarrow \mathrm{ipsiACC}}$ neuron induced by 200 ms red light stimulations (**c**). **d** Schematic of AAV (Chronos-GFP) injection into the left ACC with the retrograde transduction strategy for eNpHR-YFP expression in CLA$^{\rightarrow \mathrm{ipsiACC}}$ neurons (*left*), and optical fiber implantation and *in vivo* unit recording from the right ACC of head-fixed awake mice (*middle* and *right*). **e, f** Representative Spectrograms (SGs, *top*) and traces of the LFP signals (*below*) modulated by the blue light stimulations without (**e,** control) or during red light inhibition (**f**). The timing of light-stimulation was indicated as blue dashed lines in each trace (2 Hz, 4 times; 5 Hz, 10 times; 10 Hz, 20 times; 20 Hz, 40 times). **g** Summary of EEG delta power differences between before (pre) and during light stimulation in right ACC in conditions without (control) or with red light inhibition (n = 12, for 2 s each, two-way repeated measures ANOVA with Bonferroni's multiple comparisons test). Error bars show the SEM.

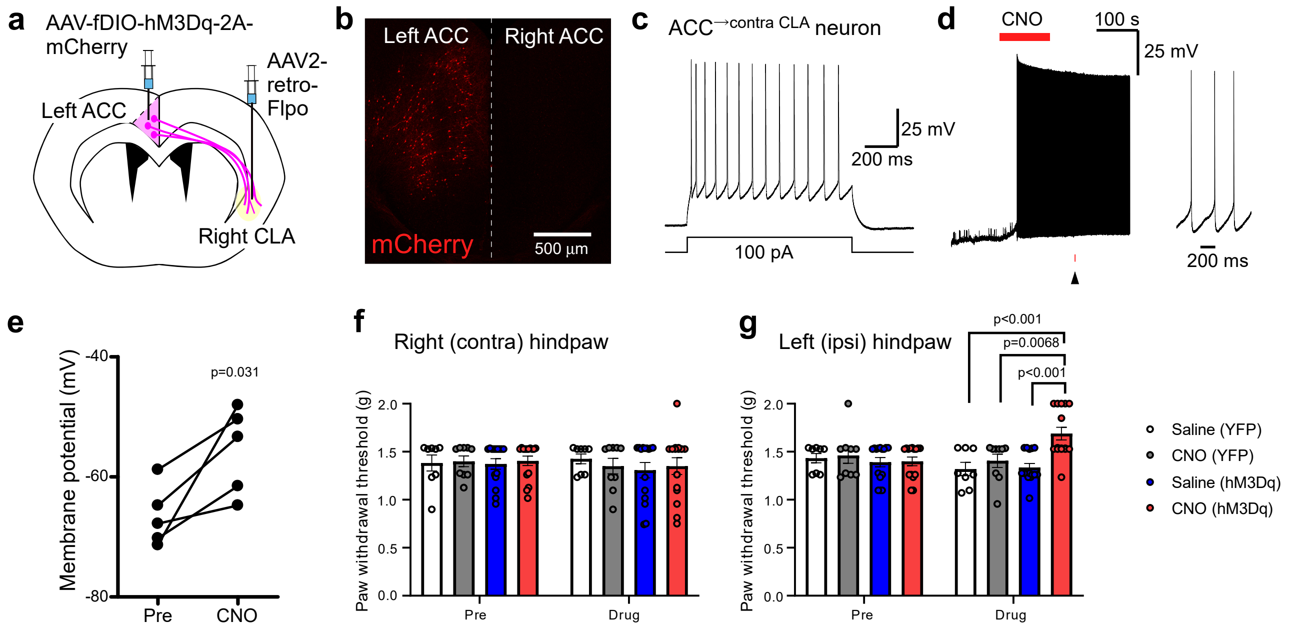
a
AAV-fDIO-hM3Dq-2A-mCherry
AAV2-retro-Flpo
Left ACC
Right CLA
b
Left ACC
Right ACC
mCherry
500 µm
c
ACC→contra CLA neuron
25 mV
200 ms
100 pA
d
CNO
100 s
25 mV
200 ms
e
Membrane potential (mV)
p=0.031
Pre
CNO
f
Right (contra) hindpaw
Paw withdrawal threshold (g)
Pre
Drug
g
Left (ipsi) hindpaw
p<0.001
p=0.0068
p<0.001
Saline (YFP)
CNO (YFP)
Saline (hM3Dq)
CNO (hM3Dq)

Supplementary Figure 6. Effects of chemogenetic activation of ACC$^{\rightarrow contraCLA}$ neurons on withdrawal threshold in naïve animals.

a Schematic of a retrograde transduction strategy for hM3Dq expression in ACC$^{\rightarrow contraCLA}$ neurons. **b** A representative image of hM3Dq-mCherry-expressing neurons in the left ACC (mCherry, red). **c, d** Example traces of firing pattern of a hM3Dq-mCherry-expressing ACC neuron (**c**) and AP discharge elicited by CNO (**d**). APs in the period indicated by arrowhead are shown in an expanded time course. **e** Quantification of CNO effect on membrane potential of hM3Dq-mCherry-expressing neurons (n = 5, two-tailed paired *t*-test). **f, g** Effects of CNO administration on withdrawal thresholds for the right and left (contralateral and ipsilateral to the left hM3Dq-mCherry- or YFP-expressing ACC neurons) (saline (YFP), n = 8; CNO (YFP), n = 9; saline (hM3Dq), n = 15; CNO (hM3Dq), n = 15, two-way repeated measures ANOVA with Bonferroni's multiple comparisons test). Error bars show the SEM.

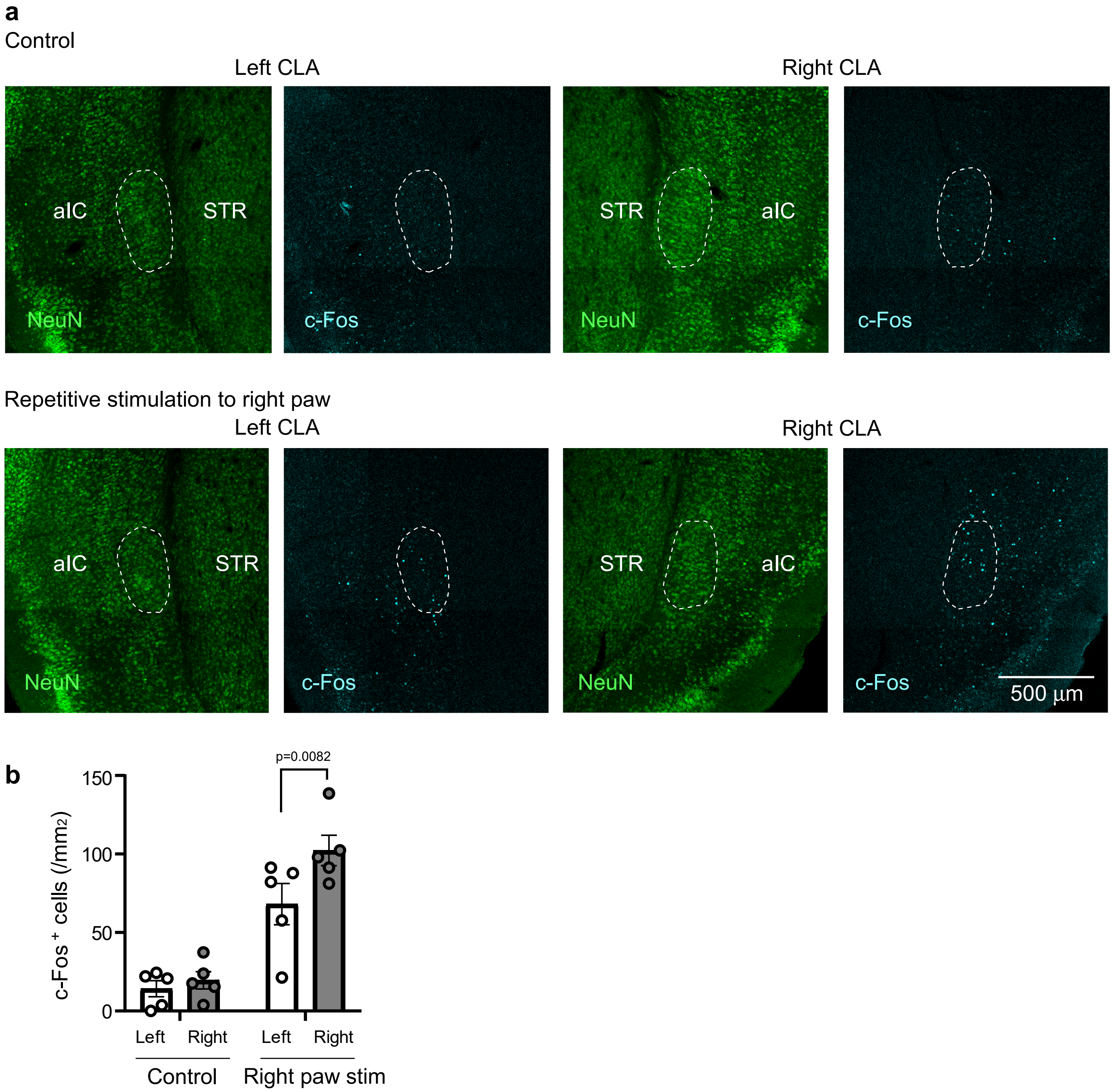

a
Control
Left CLA
Right CLA
aIC
STR
NeuN
c-Fos
STR
aIC
NeuN
c-Fos
Repetitive stimulation to right paw
Left CLA
Right CLA
aIC
STR
NeuN
c-Fos
STR
aIC
NeuN
c-Fos
500 µm
b
p=0.0082
150
100
50
0
c-Fos + cells (/mm2)
Left
Right
Left
Right
Control
Right paw stim

Supplementary Figure 7. Right paw repetitive stimulation induced a greater number of c-Fos expression in the right (ipsilateral) CLA compared to the left side.

a Representative images of c-Fos expression in the left and right CLA of mice control (without stimulation, upper panels) and after (lower panels) the repetitive stimulation applied to the right hindpaw to induce attending behaviors. c-Fos (cyan), NeuN (green), striatum (STR), anterior insular cortex (aIC) **b** Quantification of the number of c-Fos positive cells in the left and right CLA in control and after the right paw repetitive stimulation (n = 5, two-way repeated measures ANOVA with Bonferroni's multiple comparisons test). Error bars show the SEM.

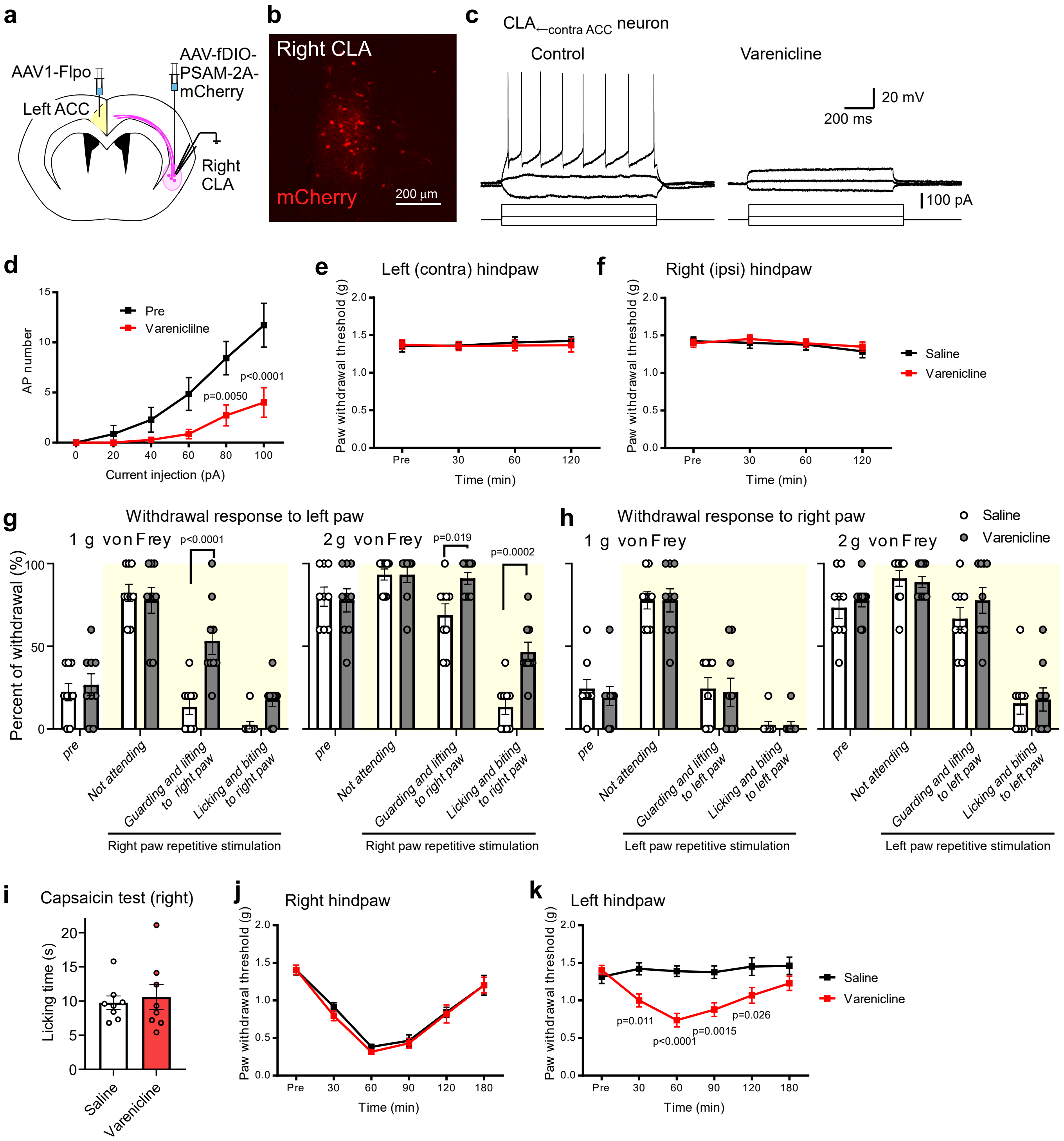

a
AAV1-Flpo
AAV-fDIO-PSAM-2A-mCherry
Left ACC
Right CLA
b
Right CLA
mCherry
200 μm
c
CLA←contra ACC neuron
Control
Varenicline
20 mV
200 ms
100 pA
d
Pre
Varenicline
AP number
Current injection (pA)
p=0.0050
p<0.0001
e
Left (contra) hindpaw
f
Right (ipsi) hindpaw
Paw withdrawal threshold (g)
Time (min)
Saline
Varenicline
g
Withdrawal response to left paw
1 g von Frey
2 g von Frey
p<0.0001
p=0.019
p=0.0002
Percent of withdrawal (%)
pre
Not attending
Guarding and lifting to right paw
Licking and biting to right paw
Right paw repetitive stimulation
h
Withdrawal response to right paw
Saline
Varenicline
Guarding and lifting to left paw
Licking and biting to left paw
Left paw repetitive stimulation
i
Capsaicin test (right)
Licking time (s)
Saline
Varenicline
j
Right hindpaw
k
Left hindpaw
p=0.011
p<0.0001
p=0.0015
p=0.026

Supplementary Figure 8. CLA$_{\leftarrow \mathrm{contraACC}}$ neurons are essential for attending behavior-induced contralateral pain modulation.

a, b Schematic of anterograde trans-synaptic tracing strategy to express PSAM^{4}-GlyR-2A-mCherry in the right CLA$_{\leftarrow \mathrm{contraACC}}$ neurons (**a**) and a representative image showing the mCherry-expressing neurons in the CLA (red) (**b**). **c** Example traces showing effect of varenicline (100 nM) on APs in response to current injections applied through the recording electrode in mCherry-expressing neurons. **d** Quantification of the effect of varenicline on relationship AP firing number and the amplitude of current injections in mCherry-expressing neurons (n = 7, two-way repeated measures ANOVA with Bonferroni's multiple comparisons test). **e, f** Effect of varenicline administration on paw withdrawal threshold for left (**e**) and right (**f**) (contralateral and ipsilateral to the PSAM^{4}-GlyR expressing CLA side, respectively) (saline, n = 9; varenicline, n = 9, two-way repeated measures ANOVA). **g, h** Varenicline effect on withdrawal rates of the left (contralateral to the PSAM^{4}-GlyR expressing CLA side) hindpaw to von Frey stimulations in control and during attending behaviors to the right hindpaw (**g**: n = 9, two-way repeated measures ANOVA with Bonferroni's multiple comparisons test) and of the right (ipsilateral to the PSAM^{4}-GlyR expressing CLA side) hindpaw to von Frey stimulations in control and during left hindpaw attending behavior (**h**: n = 9, two-way repeated measures ANOVA with Bonferroni's multiple comparisons test). **i–k** Effect of varenicline administration on capsaicin-induced nocifensive behaviors (licking and biting time) (**i**: n = 8, two-tailed unpaired *t*-test) and on capsaicin-induced mechanical hypersensitivity in the right paw (**j**: capsaicin injected side, ipsilateral to the PSAM^{4}-GlyR-expressing CLA side) and mechanical threshold in the left paw (**k**: opposite side to capsaicin injection, contralateral to the PSAM^{4}-GlyR-expressing CLA side) (n = 8, two-way repeated measures ANOVA with Bonferroni's multiple comparisons test).

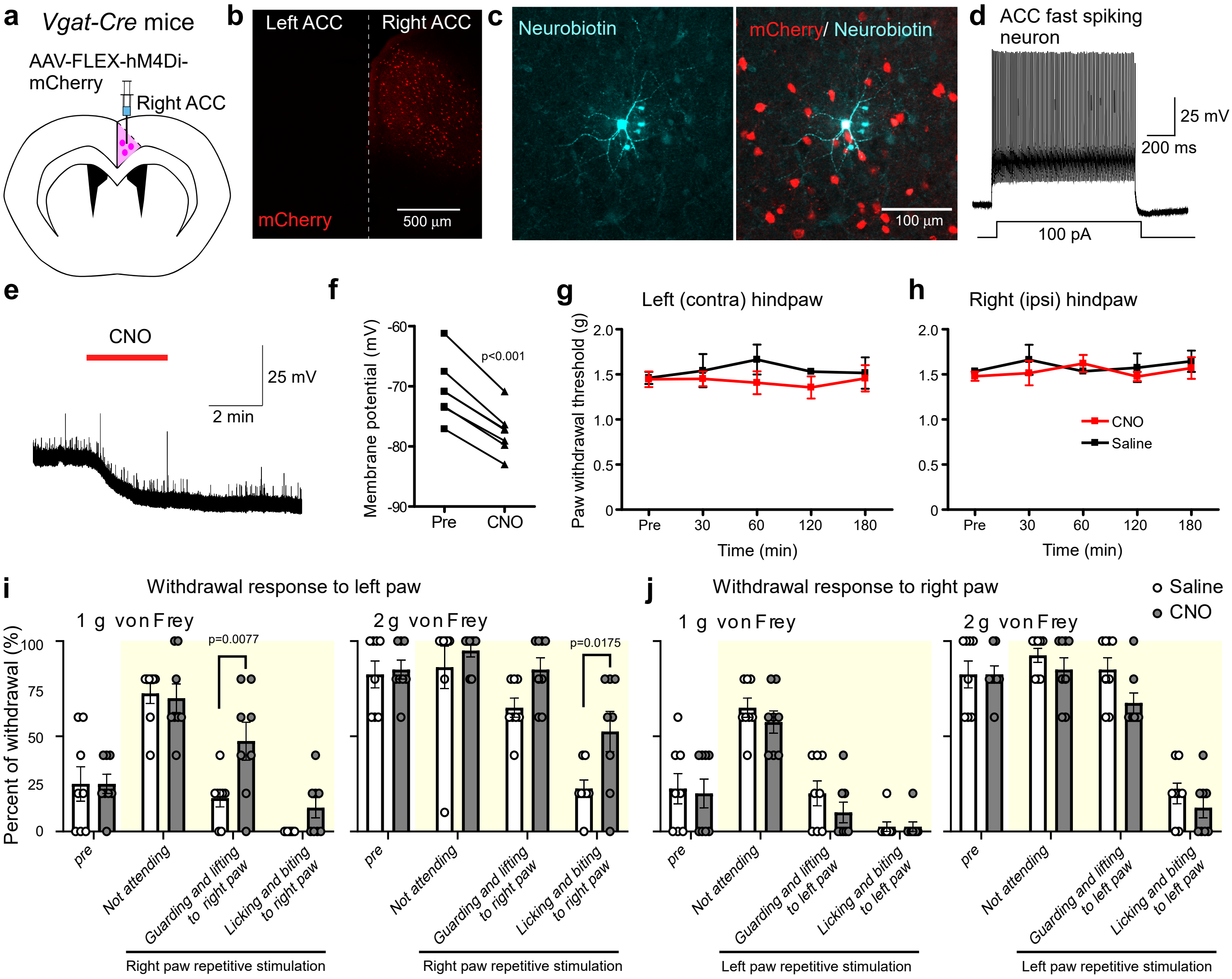
a
Vgat-Cre mice
AAV-FLEX-hM4Di-mCherry
Right ACC
b
Left ACC
Right ACC
mCherry
500 µm
c
Neurobiotin
mCherry/ Neurobiotin
100 µm
d
ACC fast spiking neuron
25 mV
200 ms
100 pA
e
CNO
25 mV
2 min
f
Membrane potential (mV)
p<0.001
Pre
CNO
g
Left (contra) hindpaw
Paw withdrawal threshold (g)
Time (min)
h
Right (ipsi) hindpaw
CNO
Saline
Time (min)
i
Withdrawal response to left paw
1 g von Frey
2 g von Frey
p=0.0077
p=0.0175
Percent of withdrawal (%)
pre
Not attending
Guarding and lifting to right paw
Licking and biting to right paw
Right paw repetitive stimulation
j
Withdrawal response to right paw
Saline
CNO
Guarding and lifting to left paw
Licking and biting to left paw
Left paw repetitive stimulation

Supplementary Figure 9. ACC inhibitory interneurons are essential for attending behavior-induced contralateral pain modulation.

a Schematic of ACC injection to express hM4Di in inhibitory interneuron of the right ACC of *Vgat-Cre* mice. **b** A representative image of hM4Di-mCherry expression in the right ACC (mCherry, red). **c–e** Representative images of neurobiotin-stained morphological feature (**c**), firing pattern (**d**, identified as fast-spiking type) and CNO-induce hyperpolarization (**e**) of mCherry-expressing ACC neurons recorded with slice patch-clamp recordings. **f** Quantification of effect of CNO application on membrane potentials in mCherry-expressing ACC neurons (n = 7, two-tailed paired t-test). **g, h** Effect of CNO administration on paw withdrawal threshold for left (**g**) and right (**h**) (contralateral and ipsilateral to hM4Di expressing-ACC side, respectively) (saline, n = 4; CNO, n = 5, two-way repeated measures ANOVA with Bonferroni's multiple comparisons test). **i, j** CNO effect on withdrawal rates of the left (contralateral to the hM4Di-expressing ACC side) hindpaw to von Frey stimulations in control and during attending behaviors to the right hindpaw (**i**: n = 8, two-way repeated measures ANOVA with Bonferroni's multiple comparisons test) and of the right (ipsilateral to the hM4Di-expressing ACC side) hindpaw to von Frey stimulations in control and during left hindpaw attending behavior (**j**: n = 8, two-way repeated measures ANOVA with Bonferroni's multiple comparisons test) .

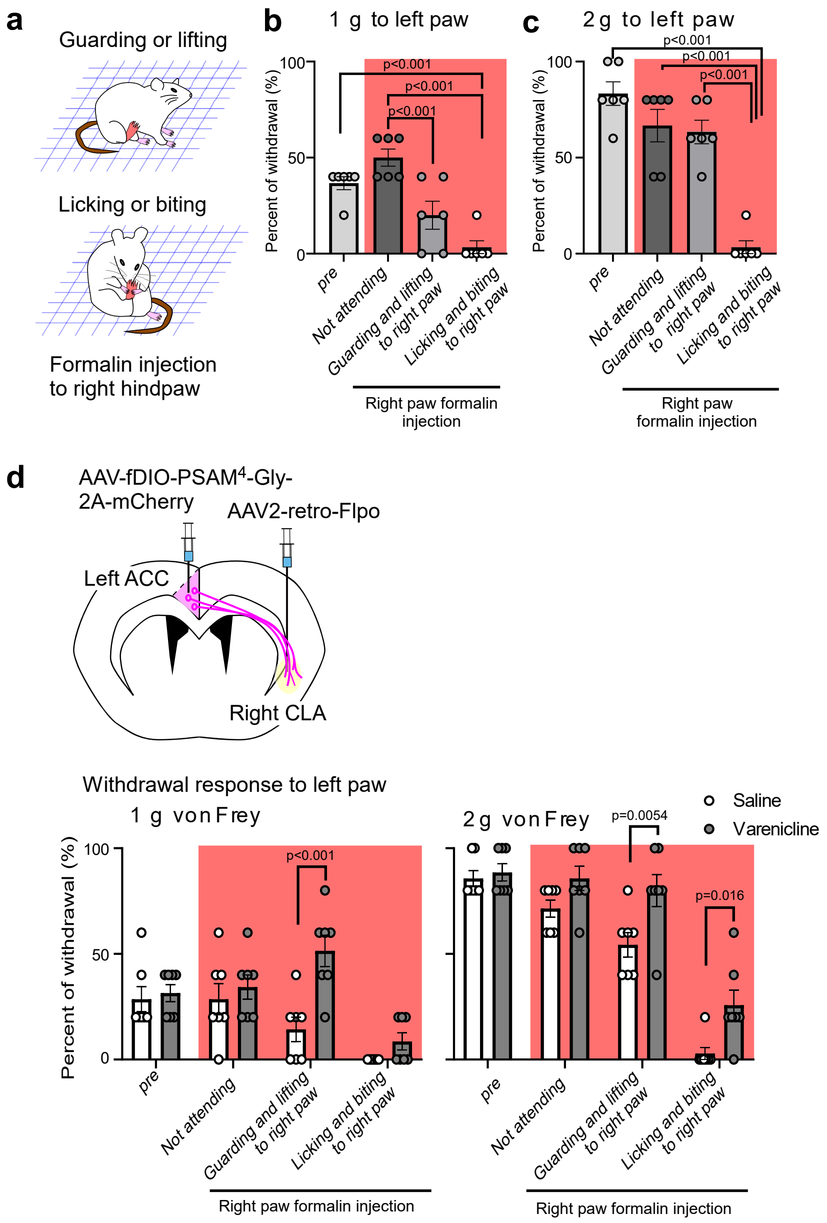
a
Guarding or lifting
Licking or biting
Formalin injection
to right hindpaw
b
1 g to left paw
Percent of withdrawal (%)
100
50
0
p<0.001
p<0.001
p<0.001
pre
Not attending
Guarding and lifting to right paw
Licking and biting to right paw
Right paw formalin injection
c
2 g to left paw
Percent of withdrawal (%)
p<0.001
p<0.001
p<0.001
Right paw formalin injection
d
AAV-fDIO-PSAM4-Gly-2A-mCherry
AAV2-retro-Flpo
Left ACC
Right CLA
Withdrawal response to left paw
1 g von Frey
2 g von Frey
Saline
Varenicline
Percent of withdrawal (%)
p<0.001
p=0.0054
p=0.016
Right paw formalin injection
Right paw formalin injection

Supplementary Figure 10. ACC$^{\rightarrow \mathrm{contraCLA}}$ neurons are important for nocifensive behavior-induced contralateral pain modulation by formalin injection.

a Mice showing attending behaviors to the right formalin-injected hindpaw. **b, c** Withdrawal rates of the left hindpaw to von Frey stimulations in control and during attending behaviors to the right hindpaw (**b**: 1 g von Frey; **c**: 2 g von Frey; n = 6, one-way repeated measures ANOVA with Tukey's multiple comparisons test). **d** Schematic of retrograde transduction strategy of PSAM^4-GlyR expression in left ACC$^{\rightarrow \mathrm{contraCLA}}$ neurons (*top*). Varenicline effects on withdrawal rates of the left (ipsilateral to PSAM^4-Gly expressing ACC side) hindpaw to von Frey stimulations in control and during attending behaviors to the right hindpaw (*bottom*, n = 7, two-way repeated measures ANOVA with Bonferroni's multiple comparisons test).
