# Supplementary material for: Anterior cingulate cross-hemispheric inhibition via the claustrum resolves painful sensory conflict
Source: Commun Biol. 2024 Mar 15;7:330. doi: 10.1038/s42003-024-06008-9 (PMC10943010; doi:10.1038/s42003-024-06008-9)
Supplement: Supplementary file 3 — Description of Additional Supplementary Files [file 42003_2024_6008_MOESM3_ESM.pdf]

Description of Additional Supplementary Files

File name: Supplementary Data

Description: The source data behind the graphs in the paper
