# Supplementary material for: Anterior cingulate cross-hemispheric inhibition via the claustrum resolves painful sensory conflict
Source: Commun Biol. 2024 Mar 15;7:330. doi: 10.1038/s42003-024-06008-9 (PMC10943010; doi:10.1038/s42003-024-06008-9)
Supplement: Supplementary file 5 — Reporting Summary [file 42003_2024_6008_MOESM5_ESM.pdf]

nature portfolio

Corresponding author(s): Hidemasa Furue, Keisuke Koga

Last updated by author(s): Feb 12, 2024

Reporting Summary

Nature Portfolio wishes to improve the reproducibility of the work that we publish. This form provides structure for consistency and transparency in reporting. For further information on Nature Portfolio policies, see our Editorial Policies and the Editorial Policy Checklist.

Statistics

For all statistical analyses, confirm that the following items are present in the figure legend, table legend, main text, or Methods section.

n/a	Confirmed	
☐	☒	The exact sample size (n) for each experimental group/condition, given as a discrete number and unit of measurement
☐	☒	A statement on whether measurements were taken from distinct samples or whether the same sample was measured repeatedly
☐	☒	The statistical test(s) used AND whether they are one- or two-sided<br>*Only common tests should be described solely by name; describe more complex techniques in the Methods section.*
☒	☐	A description of all covariates tested
☐	☒	A description of any assumptions or corrections, such as tests of normality and adjustment for multiple comparisons
☐	☒	A full description of the statistical parameters including central tendency (e.g. means) or other basic estimates (e.g. regression coefficient) AND variation (e.g. standard deviation) or associated estimates of uncertainty (e.g. confidence intervals)
☐	☒	For null hypothesis testing, the test statistic (e.g. F, t, r) with confidence intervals, effect sizes, degrees of freedom and P value noted<br>*Give P values as exact values whenever suitable.*
☒	☐	For Bayesian analysis, information on the choice of priors and Markov chain Monte Carlo settings
☒	☐	For hierarchical and complex designs, identification of the appropriate level for tests and full reporting of outcomes
☒	☐	Estimates of effect sizes (e.g. Cohen's d, Pearson's r), indicating how they were calculated

Our web collection on statistics for biologists contains articles on many of the points above.

Software and code

Policy information about availability of computer code

Data collection	MultiClamp 700A (Molecular Devices) to control Axopatch 700A amplifier<br>pCLAMP 8.2 (Molecular Devices) for digitizing and acquirement of electrophysiological data.<br>Zen 2012 (version 8.1.9.484, Carl Zeiss) for acquisition of the fluorescent image
Data analysis	Offline Sorter V3 (Plexon) for spike sorting of unit activity and LabChart8 (AD Instruments, version 8.1.16) for analyzing LFP and EMG data.<br>Clampfit (Molecular Devices, version 10.7.0.3) for analyzing slice electrophysiological data.<br>Graph Pad Prism 9 (Graph Pad, CA) for statistical analyses<br>ImageJ Fiji (ImageJ 1.52p, National Institutes of Health, https://fiji.sc) for quantification of fluorescent intensity and counting targeting cells.

For manuscripts utilizing custom algorithms or software that are central to the research but not yet described in published literature, software must be made available to editors and reviewers. We strongly encourage code deposition in a community repository (e.g. GitHub). See the Nature Portfolio guidelines for submitting code & software for further information.

Data

Policy information about availability of data

All manuscripts must include a data availability statement. This statement should provide the following information, where applicable:

- Accession codes, unique identifiers, or web links for publicly available datasets
- A description of any restrictions on data availability
- For clinical datasets or third party data, please ensure that the statement adheres to our policy

All the original data that support the findings of this study are available from the corresponding author upon requests. Source data files are provided with the manuscript.

Human research participants

Policy information about studies involving human research participants and Sex and Gender in Research.

Reporting on sex and gender	n/a
Population characteristics	n/a
Recruitment	n/a
Ethics oversight	n/a

Note that full information on the approval of the study protocol must also be provided in the manuscript.

Field-specific reporting

Please select the one below that is the best fit for your research. If you are not sure, read the appropriate sections before making your selection.

☒ Life sciences ☐ Behavioural & social sciences ☐ Ecological, evolutionary & environmental sciences

For a reference copy of the document with all sections, see nature.com/documents/nr-reporting-summary-flat.pdf

Life sciences study design

All studies must disclose on these points even when the disclosure is negative.

Sample size	No statistical methods were used to predetermine sample sizes, but the sample size was determined based on our experience with the experimental models and our reported papers (Kohro et al., Nat Neurosci, 2021; Matsumoto et al., Biochem Biophys Res Commun, 2021)
Data exclusions	Data were not excluded except in cases of viral vector or retrograde tracer misplacement. For opto-tag experiments, we excluded the neurons exhibiting higher frequency firings in the baseline state than 20 Hz.
Replication	All experiments has been successfully repeated at least two times.
Randomization	Mice were randomly grouped in all experiments.
Blinding	Researchers were not blinded during the study because limited staffs perform the particular studies, and the person handling mice or sample usually perform the assay.

Reporting for specific materials, systems and methods

We require information from authors about some types of materials, experimental systems and methods used in many studies. Here, indicate whether each material, system or method listed is relevant to your study. If you are not sure if a list item applies to your research, read the appropriate section before selecting a response.

Materials & experimental systems

n/a	Involved in the study
	☒ Antibodies
	☒ Eukaryotic cell lines
☒	Palaeontology and archaeology
	☒ Animals and other organisms
☒	Clinical data
☒	Dual use research of concern

Methods

n/a	Involved in the study
☒	ChIP-seq
☒	Flow cytometry
☒	MRI-based neuroimaging

Antibodies

Antibodies used

Polyclonal rabbit anti-c-Fos (sc-52, 1:500, Santa Cruz)
Polyclonal chicken anti-NeuN (266 006, 1:1000, Synaptic Systems)
polyclonal rabbit anti-RFP (PM005, 1:500, MBL Life Sciences)
Donkey anti-rabbit Alexa Fluor 647 (AB_2492288, 1:500, Jackson ImmunoResearch)
Donkey anti-chicken Alexa Fluor 488 (AB_2340375, 1:1000, Jackson ImmunoResearch)

Validation

All primary antibodies used in this study are validated by the manufacturer and also validated by following previous reports.
anti-c-Fos: Koga et al., Sci Rep 7 (1): 4739 (2017)
anti-NeuN: Yamada et al., J Neurosci 38 (39): 8496-8513, (2018)
anti-RFP: Hattori et al., Nat Commun 11 (1): 1631 (2020)
Secondary antibodies were widely used.

Eukaryotic cell lines

Policy information about cell lines and Sex and Gender in Research

Cell line source(s)

HEK293Twas obtained from ATCC.

Authentication

HEK293Twas authenticated by ATCC, and no further authentication was performed for this cell line in this study.

Mycoplasma contamination

HEK293Twas negative to mycoplasm contamination.

Commonly misidentified lines
(See ICLAC register)

No commonly misidentified cell lines were used in the study.

Animals and other research organisms

Policy information about studies involving animals; ARRIVE guidelines recommended for reporting animal research, and Sex and Gender in Research

Laboratory animals

Male C57BL/6J mice (CLEA Japan), and Vgat-Cre mice (B6J-Slc32a1tm2(cre)lowl/MwarJ, Stock No: 028862, The Jackson Laboratory) were used. All mice used were 8–12 weeks old at the start of each experiment and were housed at 22 ± 1 °C with a 12-h light–dark cycle with food and water ad libitum. All animals were housed in standard polycarbonate cages in groups of same-sex littermates.

Wild animals

We did not use wild animals.

Reporting on sex

In this study, we used only male animals because of the limitation of the space for maintaining and breeding both sexes of mice.

Field-collected samples

We did not use field-collected samples.

Ethics oversight

All animal studies were reviewed and approved by the Institutional Animal Care and Use Committee of Hyogo Medical University and were performed in accordance with the institutional guidelines for animal experiments and were consistent with the ethical guidelines of the International Association for the Study of Pain.

Note that full information on the approval of the study protocol must also be provided in the manuscript.
